# Case report: Pharmacokinetics of pembrolizumab in a patient with stage IV non–small cell lung cancer after a single 200 mg administration

**DOI:** 10.3389/fonc.2022.960116

**Published:** 2023-01-11

**Authors:** Fenna de Vries, Adrianus A. J. Smit, Gertjan Wolbink, Annick de Vries, Floris C. Loeff, Eric J. F. Franssen

**Affiliations:** ^1^ Department of Pharmacy, OLVG Hospital, Amsterdam, Netherlands; ^2^ Department of Pulmonary Medicine, OLVG Hospital, Amsterdam, Netherlands; ^3^ Department of Rheumatology, Amsterdam Rheumatology and Immunology Centre, Amsterdam, Netherlands; ^4^ Diagnostic Services, Sanquin Health Solutions, Amsterdam, Netherlands

**Keywords:** pembrolizumab, immunotherapy, immune-related adverse events, programmed cell death protein 1 (PD-1), pharmacodynamic (PD), pharmacokinetic (PK)

## Abstract

**Background:**

Pembrolizumab is a well-tolerated biologic agent with a potentially stable and durable anti-tumor response. Unfortunately, discontinuation of therapy can occur as a consequence of immune-related adverse effects (irAEs). These irAEs appear independent of dose and exposure. However, such irAEs might also result from pembrolizumab’s highly specific mechanism of action and current dosing regimens. However, the currently available pharmacokinetic (PK) and pharmacodynamic (PD) data to reassess dosing strategies are insufficient.

To highlight the importance of additional PK/PD studies, we present a case describing the complexity of pembrolizumab’s PK/PD after a single 200 mg pembrolizumab dose in a treatment-naive patient with non–small cell lung cancer (NSCLC).

**Case description:**

A 72-year-old man with stage IV NSCLC presented hepatotoxic symptoms 19 days after receiving the first 200 mg pembrolizumab dose. Hence, pembrolizumab therapy was paused, and prednisolone therapy was initiated, which successfully inhibited the toxic effect of pembrolizumab. However, repeated flare-ups due to prednisolone tapering suggest that the toxic effect of pembrolizumab outlasts the presence of pembrolizumab in the bloodstream. This further suggests that the T-cell–mediated immune response outlasts the programmed cell death protein 1 (PD-1) receptor occupancy by pembrolizumab, which challenges the need for the current fixed-interval strategies and their stop criteria.

Furthermore, a validated ELISA quantified pembrolizumab levels in 15 samples within 123 days after administration. A shift in the pembrolizumab clearance rate was evident ensuing day 77 (0.6 µg/mL) after administration. Pembrolizumab levels up to day 77 (9.1–0.6 µg/mL) strongly exhibited a linear, first-order clearance (R^2^ = 0.991), whereas after day 77, an accelerated non-linear clearance was observed. This transition from a linear to non-linear clearance was most likely a result of full target receptor saturation to non-full target receptor saturation, in which the added effect of target-mediated drug disposition occurs. This suggests that pembrolizumab’s targets were fully saturated at levels above 0.6 µg/mL, which is 43 to 61 times lower than the steady-state trough levels (C_trough,ss_) of the currently registered fixed-dosing regimens (3–5).

## Introduction

Pembrolizumab is a humanized immunoglobulin G4 monoclonal antibody, highly selective for programmed cell death protein 1 (PD-1). PD-1 expressed on activated T cells and this biologic agent aim to remove the immunosuppressive effect resulting from the engagement of PD-1 by programmed cell death ligands 1 or 2 (PD-L1/PD-L2) expressed on tumor cells, resulting in a stable and durable anti-tumor response in a subset of patients (1, 2). However, as an undesired effect, the blockage of PD-1 may result in unrestrained T-cell–mediated immune activation, manifesting as severe immune-related adverse effects (irAEs). According to the literature, approximately 10% of the occurring irAEs lead to (temporary) discontinuation of therapy (3–5).

However, the incidence and severity of irAEs appear unrelated to the pembrolizumab exposure at doses of 2 to 10 mg/kg per 3 weeks. The absence of an exposure–toxicity relation may be partly the consequence of pembrolizumab’s highly selective PD-1 inhibition, thereby limiting off-target toxicity. In addition, the toxic effect from on-target but undesired immune activation may not have been evident in clinical trials executed with pembrolizumab doses equal to or higher than the currently registered dose due to the full target receptor saturation throughout the study, resulting in a maximum toxic effect at all dose levels (6, 7). This full target receptor saturation throughout the dosing interval implicates the possibility of excess pembrolizumab in the bloodstream, which raises the question of whether pembrolizumab is being overdosed.

Considering the vastly increasing treatment cost using pembrolizumab and the financial burden among patients (in the Netherlands: € 29 million per year in 2016 versus €210 million per year in 2020), overdosing becomes even more highly undesirable (8–11). Alternative dosing strategies such as concentration-based dosing, dose banding, and weight-based dosing are suggested to be more cost-efficient than the current fixed-dose strategy (12–14). Therefore, it is vital to assess these alternative dosing strategies to lower the financial toxicity among patients being treated with pembrolizumab and warrant the accessibility of worldwide healthcare system.

A precondition for successfully assessing pembrolizumab’s dosing strategies is to obtain robust and relevant pharmacokinetic (PK)/pharmacodynamic (PD) data. Data from the registration trials are insufficient because these data come with some difficulties. For example, traditionally, the maximum tolerated dose is used to define a new drug’s start dose, but pembrolizumab’s highly selective mechanism of action made it impossible to identify a maximum tolerated dose. Consequently, the optimal dosing regimen was determined on the basis of a combination of animal–human transposition studies, *ex vivo* and *in vitro* assays, and PK/PD translational models, which resulted in the registration of a body weight–based dose of 2 mg/kg. A few years later, a fixed dosing regimen was considered more practical because body weight weakly influences the pembrolizumab clearance, side effects are not dose-related, and tolerable range is wide. Thus, the currently used fixed-dose regimens of 200 mg per 3 weeks (200 mg Q3W) and 400 mg per 6 weeks (400 mg Q6W) were registered (7, 15–20).

The PK/PD data in these registration trials lack real-life patient data concerning the minimal effective pembrolizumab level. Therefore, we present a case describing the PK/PD in a treatment-naive patient with non–small cell lung cancer (NSCLC) who developed hepatocellular and cholestatic toxicity after a single 200 mg pembrolizumab administration. This case provides a unique, real-life insight into pembrolizumab’s complex PK/PD and highlights the importance of additional real-life PK/PD studies in patients receiving pembrolizumab therapy.

## Case description

### Diagnosis, treatment, and complications

A 72-year-old white man with a body surface area (BSA) of 2.03 m², Chronic obstructive pulmonary disease (COPD) Gold IIIC, and 45 pack years was diagnosed with a not otherwise specified stage IV NSCLC (cTxN1M1c) originating from the right lower lobe of the lung (21). Non-symptomatic metastases were observed in the right lung hilum, the fourth thoracic vertebrae (Th4), the fifth right rib (costae 5), and the ilium. A biopsy from the ilium evinced a carcinoma positive for cytokeratin 7 and negative for cytokeratin 20, prostate-specific antigen, P40, and thyroid transcriptase factor 1. The tumor was not likely related to the squamous cell lung carcinoma (pT1aN0M0 PL0 R0), removed by lobectomy from the right upper lobe of the lung in 2009 (22). Next-generation sequencing did not reveal mutations in the B-rapidly accelerated fibrosacroma (BRAF), Kirsten rat sarcoma virus (KRAS), erythroblastic leukemia viral oncogene-2 (ERBB2), and mesenchymal–epithelial transition (MET) genes. Immunohistochemistry analysis on Anaplastic lymphoma kinase (ALK) and neurotrophic tyrosine receptor kinase (NTRK) was negative, and immunohistochemistry analysis on PD-L1 (using clone 22C3) was positive in over 90% of the tumor cells. Systemic treatment with 200 mg pembrolizumab Q3W was initiated on the basis of these carcinomas’ characteristics.

**Figure 1:**
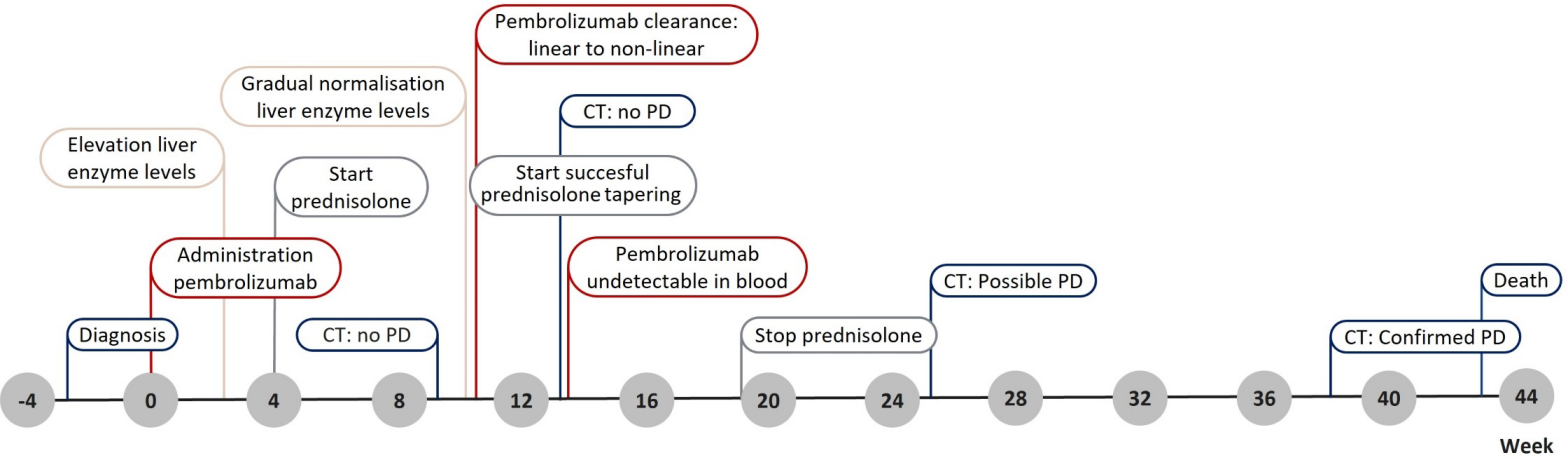
Timeline presenting the main events throughout the patient’s treatment. CT, computer tomography; PD, progression of disease.

**Figure 2:**
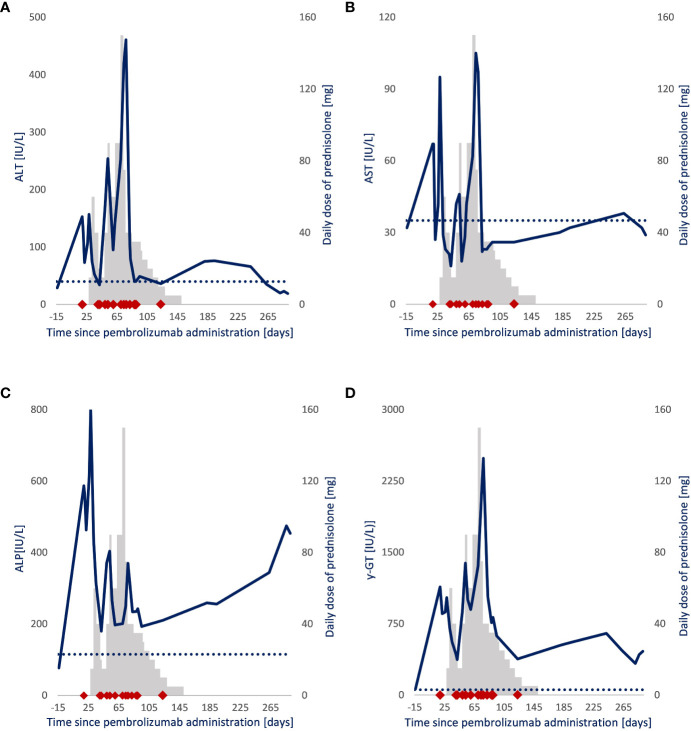
Follow-up of liver enzyme levels and daily prednisolone dose in a patient with NSCLC. Blue line: **(A)** Alanine aminotransferase (IU/L), **(B)** aspartate transaminase (IU/L), **(C)** alkaline phosphatase (IU/L), **(D)** γ-glutamyl (IU/L) transferase. Gray bars: Daily prednisolone dose (mg). ◆: Sample drawn for pembrolizumab quantification after pembrolizumab administration. **···**: Upper boundary of the liver enzymes’ normal range.

The first 200 mg pembrolizumab dose was administered 3 weeks after diagnosis (**Figure 1**). Nineteen days after administration, the patient presented with elevated levels of alanine aminotransferase (ALT), aspartate transaminase (AST), alkaline phosphatase (ALP), and γ-glutamyl (γ-GT) transferase (**Figure 2**). Additional diagnostics on day 77 after pembrolizumab administration excluded the presence of liver metastasis or any toxic or viral origin but indicated multiple liver cysts, steatosis hepatitis, and gallstone debris in the common bile duct (CBD) for which 300 mg of ursodeoxycholic acid (UDCA) three times a day was started. Furthermore, additional laboratory results indicated elevated levels of lactate dehydrogenase (LDH) from day 77 to day 95 after pembrolizumab administration (ranging from 261 to 298 IU/L, with an upper boundary of normal levels of 248 IU/L) and elevated albumin levels from day 84 to day 89 after pembrolizumab administration (ranging from 29 to 30 g/L, with reference values of 35 to 52 g/L). Meanwhile, physical controls indicated a 9% decrease in BSA to 1.86 m² between the diagnosis and 95 days after pembrolizumab administration. Nevertheless, none of these observations were the apparent cause of hepatotoxicity.

Because it could not be ruled out that the patient had a grade 2 immune-related hepatitis (ir-hepatitis) (AST/ALT = 2.5 − 5.0 × Upper limit normal value (ULN), pembrolizumab therapy was paused. Prednisolone therapy was considered but not initiated directly as developing ir-hepatitis after the first administration is uncommon (0.1%), and a spontaneous and inexplicable improvement of the levels of transaminases was observed. Nonetheless, 4 weeks after pembrolizumab administration, prednisolone therapy (1 mg/kg; 90 mg daily) was initiated when the levels abnormalized again (23). In the following 6 weeks, repeated attempts to reduce the daily prednisolone dose resulted in an immediate increase in liver enzyme levels, strengthening the idea that the patient suffered from pembrolizumab-induced ir-hepatitis. Therefore, pembrolizumab therapy was not restarted, and the patient proceeded with prednisolone therapy for over 14 weeks. Even after the discontinuation of prednisolone therapy and the start of UDCA, the cholestatic liver function never fully normalized (ALP, 454 IU/L; γ-GT, 459 IU/L).

Ten weeks after pembrolizumab administration, tumor imaging revealed no nodular abnormalities in the lower right lung; a stable costae 5 metastasis; and no axillary, hilar, or mediastinal lymphadenopathy. Similar observations were made 4 and 16 weeks later (13 and 25 weeks after pembrolizumab administration). However, the latter tumor imaging revealed a new osteoporotic compression fracture at thoracic vertebrae 8 (Th8) without specific signs of metastasis. Nearly 10 months after pembrolizumab administration, tumor imaging revealed a new lesion (either malignant or infectious) in the right lower lobe of the lung, extensive axillary and mediastinal lymphadenopathy, and extensive progression of the skeletal metastasis. Almost 4 weeks later (10 months after the 200 mg pembrolizumab administration), the patient died without apparent cause (most likely cardiac arrest during his sleep).

### Pharmacokinetics of pembrolizumab

Before administering pembrolizumab, the patient gave informed consent for an observational study in which pembrolizumab levels were quantified in residual plasma obtained for routine laboratory tests. In total, 15 samples were gathered in the days after pembrolizumab administration (respectively, on days 19, 40, 42, 49, 53, 60, 70, 74, 77, 82, 88, 89, 90, 91, and 123).

**Figure 3:**
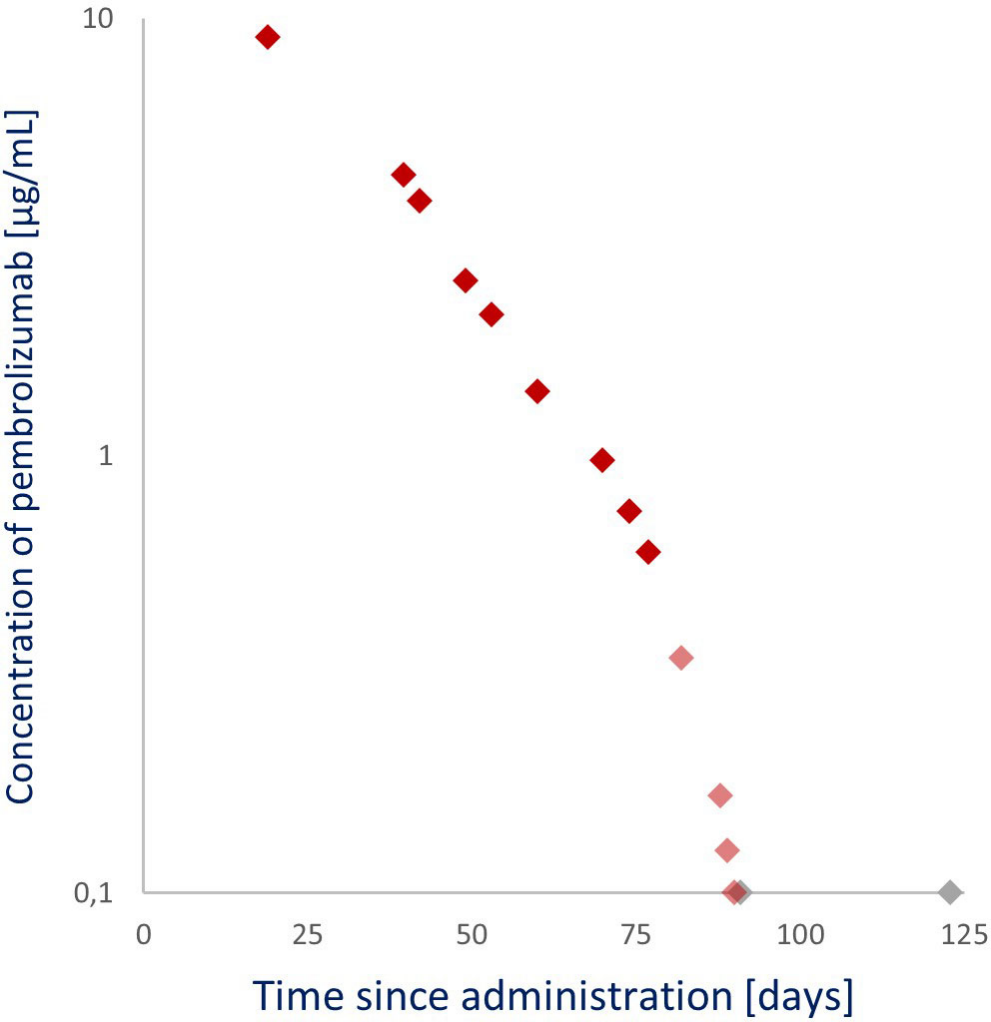
Observed pembrolizumab levels after a 200 mg per 3 weeks (Q3W) administration. ◆: Linear pembrolizumab clearance (samples from 19, 40, 42, 49, 53, 60, 70, 74, and 77 days after administration). ◆: Non-linear pembrolizumab clearance (samples from 82, 88, 89, and 90 days after administration). ◆: Below the lower limit of detection (samples from 91 and 123 days after administration).

The pembrolizumab levels of these samples were quantified using an ELISA method developed by Sanquin Diagnostics Services and validated following the U.S. Food and Drug Administration (FDA) guidelines. This method is an assay with a mean accuracy of 97% to 105% from 0.1 µg/mL (lower limit of quantification) to 200 µg/mL (upper limit of quantification) and coefficients of variation between 3.0% and 9.9%. The highest pembrolizumab level of 9.1 µg/mL, as seen in **Figure 3**, was observed in the earliest sample collected (19 days after the 200 mg pembrolizumab administration). The pembrolizumab level decreased below the assay’s lower detection limit 90 days after administration. A clear shift in clearance rate was evident from day 77 (0.6 µg/mL) after administration. Pembrolizumab levels up to and including day 77 (9.1–0.6 µg/mL) strongly exhibit a linear (first-order) clearance (R^2^ = 0.991) with a 14.6-day half-life (t_1/2_), whereas an accelerated non-linear clearance was observed after day 77.

## Discussion

The patient experienced hepatocellular and cholestatic toxicity 3 weeks after a single 200 mg pembrolizumab administration. Despite its low incidence, this toxicity was most likely related to a grade 2 ir-hepatitis (2). However, a hepatic irAE, such as ir-hepatitis, typically resolves within 6 to 9 weeks. However, in this patient, the elevated cholestatic liver enzymes (γ-GT and ALP) persisted until death (24–26). Therefore, the attribution of the patient’s comorbidities to elevated liver enzymes should be considered. An attribution to liver cysts is improbable because liver cysts rarely affect liver enzymes. Hypothetically, hepatic steatosis could cause a slight chronic elevation of ALT (approximately 100 IU/L) and γ-GT (approximately 300 IU/L). Still, on the basis of the persisting normalization of the ALT, the attribution of steatosis hepatitis is unlikely.

On the other hand, gallstone debris in the CBD can indicate an (intermittent) obstruction. Such an obstruction could contribute to the abnormality in the cholestatic liver enzymes (27–29). Although the start of UDCA therapy and the improvement in enzyme levels coincide, the extent of this attribution is inconclusive because no endoscopic retrograde cholangitis and pancreatography were performed. Nonetheless, prednisolone therapy was started because ir-hepatitis due to pembrolizumab toxicity was most likely.

The normalization of transaminases (ALT and AST) and declining cholestatic enzymes, as seen in **Figure 2**, are a consequence of the successful inhibitory effect of prednisolone on the toxic effect of pembrolizumab. Flare-ups when reducing the daily prednisolone dose suggest that the toxic effect of pembrolizumab is ongoing at that time. Moreover, this toxic effect outlasts the detectable presence of circulating pembrolizumab, suggesting that the T-cell–mediated immune response outlasts the PD-1 receptor occupancy by pembrolizumab. The occurrence of delayed immune-related events supports this hypothesis. There are published case reports of patients developing life-threatening irAEs, up to 12 months after their final pembrolizumab dose, which challenges the need for a 3- or 6-week dosing interval (23, 30–32). Nevertheless, a successful prednisolone tapering was initiated 77 days after pembrolizumab administration.

Notably, as seen in **Figure 3**, after the 77th day, pembrolizumab clearance transitioned from linear to non-linear. The pembrolizumab t_1/2_ up to that day was 14.6 days, and the quantified pembrolizumab level on that day was 0.6 µg/mL, corresponding to previously published levels where the clearance (t_1/2_, 14.1 days) transitions from linear to accelerated non-linear (0.68 µg/mL) (16–19). This linear and non-linear clearance phenomenon occurs *via* numerous physiological pathways. The predominant linear clearance occurs when the target receptor (PD-L1) is saturated, and the elimination rate is driven by proteolytic catabolism in plasma and peripheral tissues.

On the other hand, target-mediated drug disposition, a combination of linear and non-linear clearance, occurs when the target receptor is no longer saturated. Then, the elimination rate is driven by proteolytic catabolism and receptor-mediated endocytosis from the plasma or interstitium to the target cells (23, 33). Because the patient was not in a cachexic state and UDCA therapy is not expected to impact pembrolizumab’s PK, this knowledge makes it reasonable to suggest that the transition from linear clearance to a combination of linear and non-linear clearance, and thus from full receptor saturation to non-full receptor saturation, occurs close to a pembrolizumab level of 0.6 to 0.68 µg/mL, which poses the question of what concentrations far above these plasma levels add to the effectiveness of pembrolizumab.

Early *in vitro* studies described that 50% inhibition (IC_50_) of T cells reached a level of 0.535 µg/mL and maximum inhibition (I_max_) at 0.961 µg/mL (8, 11). Steady-state simulations of a post-registration study revealed a 90% probability of at least 99.31% target achievement for a 70-kg patient with a regimen of 200 mg Q3W, whereas a regimen of 1 mg/kg Q3W had a 90% probability of 96.8% target achievement. Thus, a regimen of 1 mg/kg Q3W (C_trough,ss_, 12.8 µg/mL) versus a 2 mg/kg Q3W (C_trough,ss_, 25.5 µg/mL) is predicted to only result in a modest reduction in efficacy (16, 34). Remarkably, marketing-authorization holder Merck & Co. already observed this modest reduction in efficacy 2 years earlier. They noted that no difference in the exposure–response relationship was seen across doses of 1 to 10 mg/kg and even suggested that a regimen of 1 mg/kg Q3W may be sufficient to achieve clinical efficacy. Nevertheless, the dosing strategies at 200 mg Q3W (C_trough,ss_, 36.4 µg/mL) and 400 mg Q6W (C_trough,ss_, 25.6 µg/mL) are the currently registered dosages (1, 2, 15, 34).

Unfortunately, the pharmacokinetic data from these early dose-finding studies have some difficulties. For example, defining the minimal anticipated biologic effect level is not bound to universal guidelines; wherefore, the transposition of non-human data to predict a human effect differs per study. Even so, real-world studies frequently demonstrate that clinical trial data differ from clinical practice data. Clinical trial cohorts often comprise a selective patient group that is relatively well-performing and has little to no comorbidities, whereas a real-life population is often more complex (4, 7, 35, 36). As a result, interpatient variability in pharmacokinetics was similar in trial populations but varied highly in real-life populations (16, 17, 35, 37). In conclusion, the full potential of pembrolizumab in real-life patient population is yet to be identified.

Thus, although this case report describes the PK/PD of a single patient, and an impact on the PK of pembrolizumab by covariates such as BSA, LDH, and albumin cannot be ruled out, these real-life data slightly fill existing real-life PK/PD data gap and demonstrate the complexity of pembrolizumab’s PK/PD (35). Furthermore, this case report emphasizes the need to gather additional real-life PK/PD data and explore possible alternative dosing strategies, such as concentration-guided personalized dosing strategies, to prevent overdosing on this expensive drug without compromising patient safety and drug efficacy.

## Data availability statement

The raw data supporting the conclusions of this article will be made available by the authors, without undue reservation.

## Ethics statement

The studies involving human participants were reviewed and approved by MEC-U, Nieuwegein, the Netherlands. The patients/participants provided their written informed consent to participate in this study. Written informed consent was obtained from the individual for the publication of any potentially identifiable data included in this article.

## Author contributions

FV was responsible for the database’s organization, performed the data analysis, and wrote the manuscript. FV and AS were responsible for processing the patient information. AV and FL were responsible for the sample analysis. FV, GW, and EF were responsible for the pharmacokinetic analysis. All authors contributed to the manuscript’s revision and read and approved the submitted version.
